# Adipose Tissue‐Derived Stem Cells Enhance Dermogenesis of Post‐Acne Depressed Spots

**DOI:** 10.1111/jocd.70399

**Published:** 2025-10-13

**Authors:** Afzaal Bashir, Mahmood S. Choudhery, Sunniya Afzaal, Salman Ali, Sunaina Afzaal

**Affiliations:** ^1^ Jinnah Hospital Lahore/Allama Iqbal Medical College Lahore Pakistan; ^2^ Department of Human Genetics & Molecular Biology University of Health Sciences Lahore Pakistan; ^3^ Shalamar Medical and Dental College Lahore Lahore Pakistan; ^4^ Punjab Rangers Teaching Hospital Lahore Lahore Pakistan

**Keywords:** ASCs, dermal thickness, enriched fat, lipofilling, post‐acne spots

## Abstract

**Background:**

Post‐acne depressed spots appear due to localized fat atrophy and disruption of the dermis. Surgical excision of acne spots is challenging; thus, novel management is required.

**Aims:**

The aim of this study is to compare the long‐term effects of conventional and ex vivo expanded ASCs enriched fat for the treatment of post‐acne spots.

**Patients/Methods:**

After informed consent, 40 patients having post‐acne spots on the face were recruited and, after explaining, placed in two groups depending upon their choice. In lipofilling‐only group (LF), adipose tissue was harvested, prepared, and injected intradermally and subdermally in the depressed spots. In ASC‐enriched lipofilling group (ASC‐LF), fat waɓgʻ enriched with cultured ASCs before injection. Improvement of the contour was noted clinically after 1 year. Differences in dermal thickness were confirmed histologically on punch biopsies. Evaluation of pictures was done by two consultant plastic surgeons blinded to the technique. Similarly, the satisfaction of patients was also noted.

**Results:**

Mean age of patients was 26.32 (±3.34) and 72% of patients were females. Cheeks only were involved in 57% of cases while cheeks associated with involvement of lateral forehead in 29% of cases, nose in 11% of cases, and chin in 3% of cases. In ASC‐LF group, improvement in dermal thickness (1.2 ± 0.6 mm) was higher compared to LF group (0.5 ± 0.3 mm). The mean scores of patients and physician satisfaction were higher in ASC‐LF group.

**Conclusion:**

This novel strategy significantly improved the acne‐related contours by increasing dermal thickness.

**Trial Registration:**

www.clinicaltrials.gov: NCT06135519

## 1 Introduction

Acne is one of the common skin issues in teenagers and young adults requiring proper management during the active phase as well as the post‐inflammatory stage of the disease [1]. It is managed by the use of one or various combinations of cleansers, retinoids, antibiotics, anti‐inflammatory drugs, and hormonal treatment at the active disease phase. Sometimes, it takes weeks to get the disease to settle down [2]. The active phase may result in depressed spots due to localized fat atrophy and dermal disruption by inflammation, spontaneous rupture of epithelial tissue, or because of scratching by the patient. In this post‐inflammatory stage, treatment of these acne spots becomes difficult due to many reasons [1, 2]. Firstly, surgical excision of resultant spots is difficult as their sizes are very small, and they are in a large number. Secondly, filler injection under these spots with subcision, or even without subcision, does not adequately treat the deformity. Currently, a combination of synthetic fillers or fat with resurfacing of skin by LASER, peeling, or dermabrasion is used [3]. In spite of all these modalities to adequately address post‐acne spots adequately, treatment is still awaited.

Stem cells are regenerative cells in the body with self‐renewal and differentiation capabilities. They have the potential to repair and replace damaged tissues and organs. Stem cells are found in various tissues and organs. Adipose tissue is a major autologous source of stem cells for clinical purposes. Adipose tissue is a preferred source of stem cells for regenerative medicine applications due to its relative abundance, ease of isolation, biocompatibility, and low donor morbidity. As compared to bone marrow, adipose tissue contains 500‐fold more stem cells called adipose tissue‐derived stem cells (ASCs). ASCs have identical properties to mesenchymal stem cells (MSCs) isolated from bone marrow or other sources. The role of adipose tissue has recently been evaluated in various diseases and disorders. Its importance for aesthetics cannot be denied as it is a natural filler. Adipose tissue can be used in various formats such as whole adipose tissue, nanofat, as stromal vascular fraction (SVF) or as a pure population of stem cells, that is ASCs.

The aim of this study is to compare long‐term effects of conventional fat grafting and ex vivo expanded adipose tissue‐derived stem cells (ASCs) enriched lipofilling for the treatment of post‐acne depressed spots. ASCs were isolated by enzymatic digestion of adipose tissue. ASCs were injected intradermally and sub‐dermally in depressed acne spots of patients. One year after the injection, improvement in depressed spots was assessed by comparing before and after pictures of the treated area, differences in dermal thickness, as well as patient and physician satisfaction scores. The contour treated using ASC‐enriched adipose tissue exhibited improvement in dermal thickness in concordance with high patient and physician satisfaction scores. This novel pre‐enrichment strategy is not only suitable for the treatment of acne‐related face contours but also for other similar endeavors requiring regeneration of tissues. This study has limitations of just addressing post‐acne scars only and not dealing with other types of scars for which another study should be planned.

## 2 Materials and Methods

After consideration by the Institutional Review Board vide letter IRB Ref 451/2023 dated 22nd Aug 2023, patients were explained about the types of treatment offered. Forty (40) patients having post‐acne spots on the face were recruited and informed consent was taken. Depending on their choice, the patients were placed in two groups, that is lipofilling group (LF) in which only homogenized adipose tissue was injected, and ASC‐enriched lipofilling group (ASC‐LF) in which cultured autologous ASCs enriched adipose tissue was employed. The patients of both sexes and all ages were included in the study. The patients with active chronic diseases in the last 6 months, or those using other treatments like filler, LASER, peeling, keratolytic agents, or dermabrasion were not considered for the study.

At the day of the procedure, patients were called into the operation theater, and adipose tissue was harvested by liposuction under tumescent anesthesia from the infraumbilical region using a 4 mm blunt cannula by the Colman technique. Briefly, 0.5% xylocain with 1:100 000 epinephrine was injected by tumescent technique, allowed to take adrenaline effect for 30 min, and fat was harvested by applying 2 mL suction pressure from a 10 mL syringe manually. In patients who chose lipofilling only (LF), the fat was homogenized by passing it through our own invented homogenizer (Patent with IPO, Pakistan) by pushing from a 10 mL syringe attached at one end to the 10 mL syringe attached at the other end of the homogenizer and injected intradermally and subdermally using a 27G needle in the depressed post‐acne spots. Meanwhile, in the ASC‐enriched lipofilling group (ASC‐LF), fat was extracted to harvest ASCs at the first procedure. The extracted adipose tissue was processed further in the laboratory to isolate adipose tissue‐derived stem cells (AT‐ASCs). For ASC separation, the extracted fat was washed with phosphate‐buffered saline and digested with collagenase type IV at 37°C. After 5 min, minimal essential medium was mixed in equal volume with the digested fat, and the solution was filtered through a 100 μm filter. Centrifugation at 1000 rpm was done for 10 min, and the precipitate was cultured till the fourth pass. Patients of the ASC‐LF group were called back once fourth‐pass ASCs were in hand; fat was re‐harvested with the same technique, homogenized in the same way as for the LF group, and enriched with ex vivo cultured stem cells, that is ASCs, before injecting intradermally and subdermally in the depressed spots.

Patients of both groups were observed for 1–2 h for swelling or bruising at donor or recipient areas and discharged on the same day on oral first‐generation cephalosporin and acetaminophen three times a day for 5 days. Patients were kept under monthly follow‐up for 1 year, and improvement in the contour of depressed spots was noted clinically and documented with pre‐ and post‐operative pictures.

Evaluation of pre‐ and one‐year post‐procedure pictures was performed by two consultant plastic surgeons blinded to the technique applied and was ranked by scoring (1–10). Satisfaction of each patient was also registered as highly satisfied, satisfied, and unsatisfied. Incisional biopsy was taken from lesions larger than 5 mm in which half of the lesion was biopsied, processed, and examined under a microscope after staining. Dermal thickness was measured manually on microscopic pictures by using a caliper. Similarly, post‐operative dermal thickness was again determined by incisional biopsy of the remnant lesion after one year of treatment. Two to three biopsies were taken from each patient.

### 2.1 Statistical Analysis

SPSS version 22 was utilized to calculate means, percentages, and frequencies; a paired *t*‐test was applied to determine the *p*‐value.

## 3 Results

**FIGURE 1:**
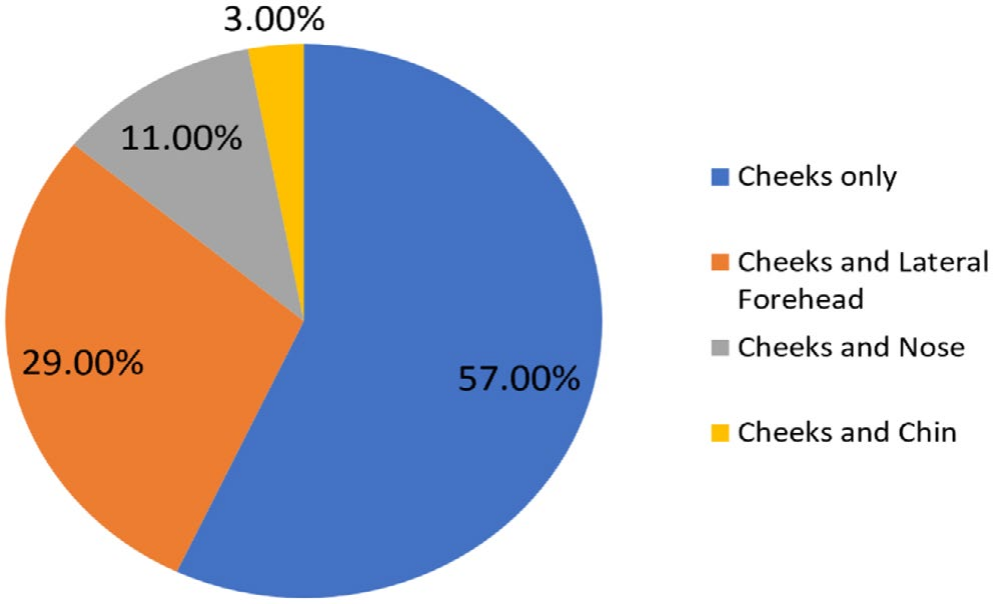
Pie chart showing face areas involved in the study for treatment.

Mean age of the patients was 26.32 (±3.34) and 72% patients were females. Cheeks were involved in all the cases presented. Involvement of cheeks only was found in 57% cases while cheeks were also having associated involvement of lateral forehead in 29% cases, nose in 11%, and chin in 3% cases as shown in Figure 1. The comparative characteristics of both populations have been presented in Table 1.

**TABLE 1:** Comparative characteristics of recruited patients in two groups.

Character	ASC‐LF	LF
Age	27.41 (±2.33)	26.82 (±3.39)
Sex		
Females	16	13
Males	4	7
Active acne duration (Years)	3 (±1.78)	2 (±3.52)
Duration of spots (Years)	2 (±3.91)	2 (±4.63)
Area involved (%)		
Cheeks only	52	59
Cheeks + Forehead	34	27
Cheeks + Nose	12	11
Cheeks + Chin	2	3

**FIGURE 2:**
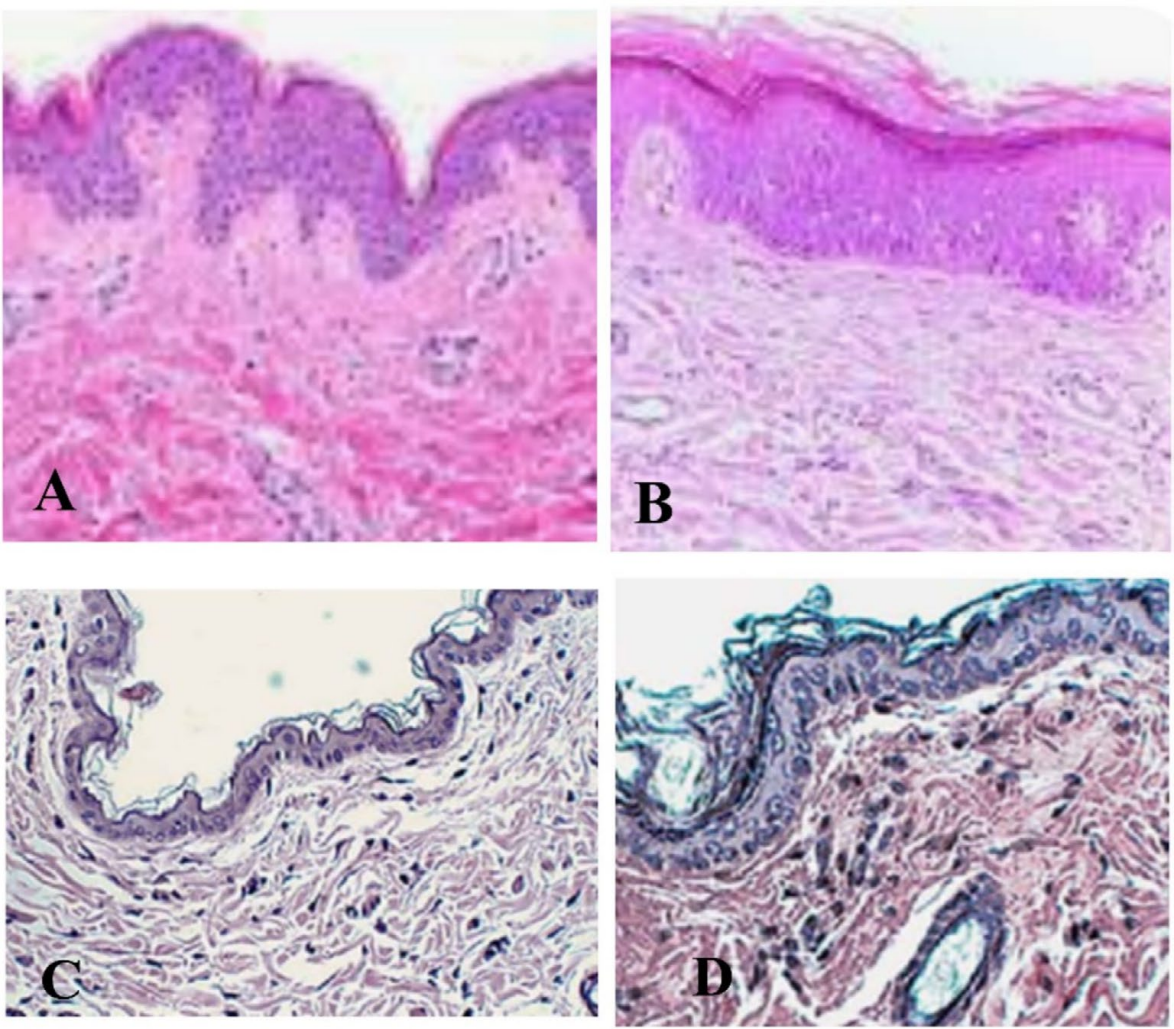
Dermal thickness in ASC‐LF and LG group. (A) shows dermal thickness before ASC‐LF treatment while (B) shows the dermal thickness 1 year after ASC‐LF application. (C) Dermal thickness before lipofilling in LF group (D) and 1 year after LF.

In ASC‐LF group, a significant improvement in dermal thickness was observed in as compared to LF group. The difference of dermal thickness pre‐injection and 1 year post‐injection was calculated by subtraction of the pre‐ and post‐procedure values. In ASC‐LF group, increase in dermal thickness was 1.2 (±0.6) mm in comparison to LF which was 0.5 (±0.3) mm after 1 year of the treatment (Figure 2). On application of *t*‐test, *p* value was found to be 0.03 which shows significance of ASC‐LF over LF group.

**FIGURE 3:**
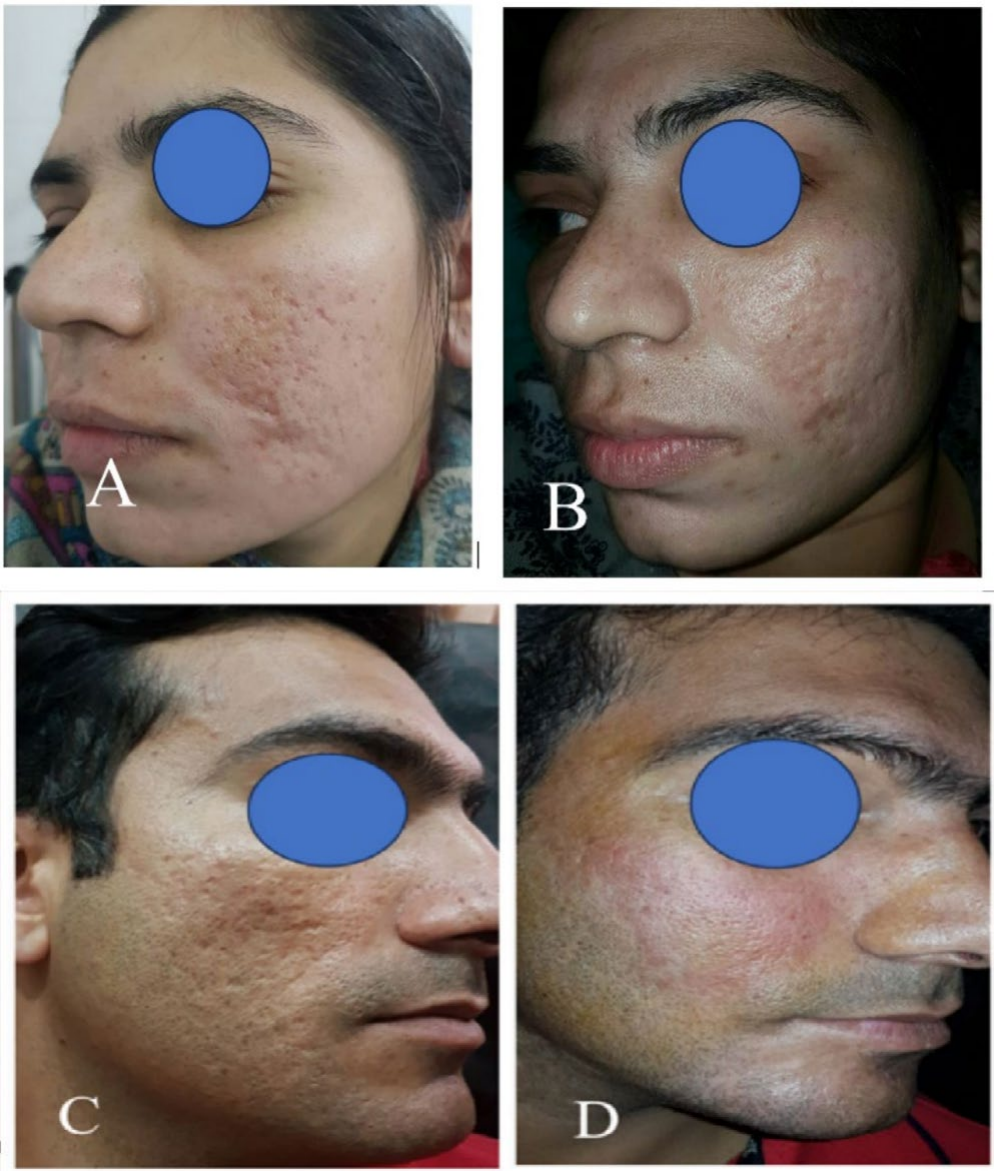
Pre‐operative and 1 year post‐operative skin appearance. (A, B) show results of ASC‐enriched lipofilling pre‐operative and 12 months post‐operative, respectively. The lipofilling‐only group shows a representative picture of a patient shown pre‐operative (C) and 1 year post‐operative (D).

Scoring on pre‐ and post‐operative pictures by two consultant surgeons was significantly high in ASC‐LF group (8.2 ± 1.3 and 7.9 ± 1.4) as compared to LF group (5.5 ± 1.7 and 4.9 ± 1.3). Patients treated with lipofilling enriched with expanded adipose stem cells were also significantly (79%) highly satisfied as compared to 49% in LFG (Figure 3).

Bruising at the donor area was found in 23% of patients, which settled within 2 weeks with olive oil massage. Needle marks at recipient areas vanished in 5 (±3.39) days in the LF group, while in 5 (±2.16) days in ASC‐LF patients. Bruising at recipient areas was completely unnoticeable in LF in 7 (±3.29) days, and in ASC‐LF subjects, it healed in 9 (±2.13).

## 4 Discussion

Mesenchymal stem cells (MSCs) are a type of adult stem cell. Initially found in bone marrow, MSCs were later identified in various other tissues as well. These cells have high proliferative and differentiation potential. Currently, several clinical trials are using MSCs for the regeneration of various tissues, such as Farabi et al. [4, 5, 6] used ASCs in the healing of wounds; Yang et al. employed stem cells in fracture management; and similarly, Bektas et al. found their utility in neuronal tissue regeneration. Adipose tissue is a versatile tissue for regenerative medicine applications. As proposed by Szychta, adipose tissue has been frequently applied as a soft tissue filler, as it is natural, biocompatible, and is available in large quantities due to its many properties, such as being autologous, having regenerative potential, and its pliability [7]. Adipose tissue can be used in different forms, such as whole adipose tissue, stromal vascular fraction, or ASCs. Adipose tissue can also be enriched with SVF or ASCs to enhance its regenerative and reparative properties. In many studies, including Meretsky et al. and even one of our own studies, it was suggested that enrichment of adipose tissue with ex vivo expanded stem cells before grafting ensures the survival of maximum adipose tissue at the recipient areas of the human body. Initially, where survival of fat at the grafted area was expected to be 40%–60%, it has increased to 80%–90% if pre‐harvested fat was used to isolate stem cells and incorporated in the fat after expansion on a culture plate [8, 9]. ASCs contained in adipose tissue have identical characteristics to MSCs. Studies indicate that ASCs can be used for cutaneous rejuvenation [10]. People are trying to find out their application in treating non‐healing skin ulcers, pigmentation, as well as longevity of fat as a filler [8, 11].

Increase in dermal thickness has been found by the application of MSCs in some studies which propose that thickness is enhanced by the change created in the ambience of that area due to cytokine production which ultimately results in promoting collagen. Due to the aforementioned effect, stem cells have found their utility in aesthetic dermatology and plastic surgery for facial rejuvenation to treat wrinkles [12]. Similarly, some studies have also described the role of ADCs in collagen production and leading to dermal repair which suggests the application of ADCs in the treatment of skin and mucosal breaks in wounds, particularly in complicated wounds such as diabetes where collagen is in the form of glycosylated collagen [7, 13]. In our study, in contrast to other studies, the break in the dermis was partial, and it was due to the inflammatory process as well as due to mechanical disruption in the acne‐involved areas [14]. Chemical fillers are traditionally used in filling the depressed spots, but they are not only on a temporary basis and resorb with time, but they also do not give a natural feel [15]. Fat, being a natural cushion, not only improves the contour but the presence of ASCs, by enrichment, plays a role in its longevity and promotes collagen synthesis [8, 16]. For post‐acne depressed spots, fat grafting enriched with MSCs is one of the promising solutions, though its use is limited so far due to the lack of expertise as well as the lack of facilities available in different centers. Our plan is to keep these patients under follow‐up as with our other studies involving ASCs for any evolution and plan to conduct more studies on this future source of modality.

## 5 Conclusion

ASC‐enriched lipofilling has a significant impact on addressing post‐acne depressed spots, not only by providing a filling effect but also by increasing the dermal thickness of the facial skin.

## Author Contributions

Afzaal Bashir conceptualized the idea. Afzaal Bashir performed the assays. Afzaal Bashir, Sunniya Afzaal, Salman Ali, and Sunaina Afzaal wrote the original version of the manuscript. Afzaal Bashir, Sunniya Afzaal, Salman Ali, and Sunaina Afzaal prepared, designed, and modified the figures and revised the manuscript. Mahmood S. Choudhery critically reviewed the manuscript. All authors reviewed the final version of the manuscript.

## Ethics Statement

After consideration by the Institutional Review Board vide letter IRB Ref 451/2023 dated 22nd Aug 2023.

## Conflicts of Interest

The authors declare no conflicts of interest.

## Data Availability

The data that support the findings of this study are available from the corresponding author upon reasonable request.
